# Respiratory Dysbiosis in Canine Bacterial Pneumonia: Standard Culture vs. Microbiome Sequencing

**DOI:** 10.3389/fvets.2019.00354

**Published:** 2019-10-11

**Authors:** Aida I. Vientós-Plotts, Aaron C. Ericsson, Hansjorg Rindt, Carol R. Reinero

**Affiliations:** ^1^College of Veterinary Medicine, University of Missouri, Columbia, MO, United States; ^2^Department of Veterinary Medicine and Surgery, College of Veterinary Medicine, University of Missouri, Columbia, MO, United States; ^3^Comparative Internal Medicine Laboratory, University of Missouri, Columbia, MO, United States; ^4^University of Missouri Metagenomics Center, University of Missouri, Columbia, MO, United States; ^5^Department of Veterinary Pathobiology, College of Veterinary Medicine, University of Missouri, Columbia, MO, United States

**Keywords:** microbiota, pneumonia, respiratory, dog, bronchoalveolar lavage, culture, sequencing, dysbiosis

## Abstract

It is unknown how the respiratory microbiome influences and is influenced by bacterial pneumonia in dogs, as culture of lung samples and not microbial sequencing guides clinical practice. While accurate identification of pathogens are essential for treatment, not all bacteria are cultivable and the impact of respiratory dysbiosis on development of pneumonia is unclear. The study purposes were to (1) characterize the lung microbiome in canine bacterial pneumonia and compare deviations in dominant microbial populations with historical healthy controls, (2) compare bacteria identified by culture vs. 16S rDNA sequencing from bronchoalveolar lavage fluid (BALF) culture-, and (3) evaluate similarities in lung and oropharyngeal (OP) microbial communities in community-acquired and secondary bacterial pneumonia. Twenty BALF samples from 15 client-owned dogs diagnosed with bacterial pneumonia were enrolled. From a subset of dogs, OP swabs were collected. Extracted DNA underwent PCR of the 16S rRNA gene. Relative abundance of operational taxonomic units (OTUs) were determined. The relative abundance of bacterial community members found in health was decreased in dogs with pneumonia. Taxa identified via culture were not always the dominant phylotype identified with sequencing. Dogs with community-acquired pneumonia were more likely to have overgrowth of a single organism suggesting loss of dominant species associated with health. Dogs with secondary bacterial pneumonia had a greater regional continuity between the upper and lower airways. Collectively, these data suggest that dysbiosis occurs in canine bacterial pneumonia, and culture-independent techniques may provide greater depth of understanding of the changes in bacterial community composition that occur in disease.

## Introduction

Canine bacterial pneumonia is a common respiratory disorder, occurring as primary disease process, or secondary to aspiration, viral infections (1, 2), immunodeficiency, or a nosocomial event (3). In both humans and companion animals, bacterial pneumonia can be life-threatening making prompt diagnosis and targeted treatment essential. Diagnostic approaches to identify the causative agents have traditionally relied on *ex vivo* culture from carefully collected airway lavage [e.g., bronchoalveolar lavage fluid (BALF)]. This method is predicated on the belief that the deep airways are largely free of bacteria and any growth on selective media represents aberrant colonization. The recent development of culture-independent molecular techniques has revealed that in humans (4), cats (5), dogs (6), sheep (7), and likely other host species, the healthy lungs harbor low biomass microbial populations seeded via direct extension from upper airway communities, repeated microaspiration, and inhalation of bacteria in air (8). Moreover, these culture-independent methods have reinforced that a lack of cultivable organisms does not necessarily indicate a sterile environment (9). Collectively, such findings suggest that sequencing methods might have clinical utility in the identification of microbes associated with bacterial pneumonia. Toward that end, the current study compared the results of traditional culture-based methods and a targeted sequencing approach applied to 20 BALF samples collected from 15 dogs affected with bacterial pneumonia in a referral veterinary hospital setting.

Canine bacterial pneumonia is categorized as either community-acquired pneumonia (CAP) or secondary bacterial pneumonia (SBP) based on the etiology, clinical presentation, and patient history. As the name implies, CAP is typified by known contagious pathogens, such as *Bordetella bronchiseptica* and *Streptococcus equi subspecies zooepidemicus* and is often seen in dogs with a history of acute onset clinical signs following exposure to reservoirs of infectious agents, such as shelters, boarding facilities, and dog parks (10, 11). Secondary bacterial pneumonia, on the other hand, occurs as a sequela to a predisposing anatomic or physiological condition, such as megaesophagus, laryngeal paralysis, or ciliary dyskinesia (12), and the microbes recovered in a diagnostic sample are often not primary contagious pathogens, *per se*. Rather, dysfunction of the upper respiratory tract or gastrointestinal tract allows or facilitates increased translocation of material to the lower airways and/or prevents effective microbial clearance, leading to the hypothesis that the lower and upper airway microbiota would be more similar in cases of SBP relative to cases of CAP. To address this question, oropharyngeal (OP) swabs were collected from a subset of dogs alongside BALF samples and the compositional similarity of OP and BALF microbiota was evaluated in the context of clinical diagnoses and predisposing anatomic factors.

## Materials and Methods

### Experimental Design

The current study was performed prospectively at the University of Missouri Veterinary Health Center (VHC), a referral and primary care veterinary hospital located in Columbia, MO, USA. All dogs contributing samples to the current study presented to the VHC with clinical signs related to bacterial pneumonia between August 2016 and December 2017. Bronchoscopic examination and diagnostic collection of BALF were performed as part of their standard care. Peripheral blood was also collected at presentation for hematologic and serum chemistry analyses. Dogs were then diagnosed with bacterial pneumonia based on clinical signs associated with septic suppurative inflammation or a positive aerobic or anaerobic culture result of BALF, and categorized by type of pneumonia based on the history, clinical signs and other diagnostic findings. Dogs were of various breeds and ages; a table showing the range of patient demographics is provided in **Table 2**.

### Sample Collection

Anesthetic protocols were performed at the discretion of a board certified veterinary anesthesiologist. Samples were collected as previously described (5). Briefly, after induction for anesthesia, while avoiding the rest of the oral cavity, a sterile swab was used to vigorously rub the caudodorsal aspect of the oropharynx, from a subset of patients (see Table 1). The swab was added to 800 μL lysis buffer adapted from Yu and Morrison (13). Dogs were initially intubated using sterile endotracheal tubes. Control samples were obtained by running a 10 ml aliquot of sterile saline through the endoscope channel before its use. Immediately prior to the bronchoscopy, the endotracheal tube was replaced with a sterile red rubber catheter to provide oxygen and the endoscope was passed directly through the larynx into the tracheobronchial tree. BALF collection was performed by instilling one or two 20 mL aliquots of sterile saline through the channel of a sterile bronchoscope when wedged in an airway. All dogs provided one BALF sample with the following exceptions: dog I provided one sample on 1/12/2017 (I1) and two samples from the left and right lung lobes in on 11/2016 (I2 and I3, respectively), dog M provided samples on 11/4 and 12/20 of 2016 (M1 and M2), and dog G provided samples on 11/11, 12/1, and 12/21 of 2016 (G1, G2, and G3, respectively). Following collection of BALF, samples were split to provide a minimum of 1 mL of material to the University of Missouri Veterinary Medical Diagnostic Laboratory for culture on Blood agar and MacConkey agar plates for aerobic cultures, and chocolate agar plates for anaerobic cultures. All aerobic samples were incubated at 35°C, and anaerobic cultures were incubated at 35°C with 95% air and 5% CO_2_ for 24–36 h. The laboratory does not culture *Mycoplasma* spp. in large part due to challenging growth requirements. Bacterial isolates were Gram-stained and identified with conventional biochemical reactions (14), the Automated Sensititre AP-80 or AP 90 for aerobic bacteria or the MALDI-TOF identification system (Matrix Assisted Laser Desorption/Ionization-Time of Flight: Bruker Daltonics, Inc. 40 Manning Road, Manning Park, Billerica, MA 01821). Aerobic susceptibility testing was performed with the Sensititre Micro-Broth (Thermofisher Scientific 12076 Santa Fe Drive, Lenexa, KS 66215) dilution minimal inhibitory concentration system. Up to 30 mL of the remaining BALF material was promptly centrifuged, and the resulting pellet was frozen and maintained at −80°C until DNA extraction was performed.

**Table 1:** Comparison of culture and targeted sequencing results for lower airways (BALF) and relative abundance of predominant OTU in BALF found in upper airways.

	**BALF**					**OP**
**Dog**	**Culture results**	**Closest 16S rRNA match**	**RA (%)**	**16S rRNA > 10% RA**	**RA (%)**	**RA (%)**
A^*^	*Streptococcus canis*	*Streptococcus canis*	99.30	*Streptococcus* spp.	99.30	10.88
B^*^	*Bordetella bronchiseptica*	Not detected		*Mycoplasma canis* PG14	53.30	7.67
				*Mycoplasma* sp. VJC358	44.60	1.72
C^*^	*Enterococcus faecalis*	Not detected		*Mycoplasma* sp.	99.60	7.87
	*Enterococcus hirae*	Not detected				
	*Lactobacillus* sp.	*Lactobacillus* sp.	<0.01			
	*Corynebacterium* sp.	*Corynebacterium* sp.	<0.01			
	*Pseudomonas* sp.	*Pseudomonas* sp.	<0.01			
D	*Streptococcus canis*	*Streptococcus* sp.	96.40	*Streptococcus* sp.	96.40	
E	*Bordetella bronchiseptica*	Not detected		*Ureaplasma* sp.	25.90	
				*Mycoplasma* sp.	15.40	
				*Pseudomonas* sp.	13.30	
				*Achromobacter xylosoxidans*	11.40	
	*Brevundimonas vesicula*	*Brevundimonas* sp.	6.80	*Bradyrhizobium* sp. T92	10.50	
F	*Staphylococcus schleiferi*	*Staphylococcus* sp.	0.20	*Acinetobacter* sp.	37.90	
				*Rhizobium* sp.	20.60	
				*Brevundimonas* sp.	11.20	
	*Bacillus* sp.	Not detected		*Bradyrhizobium* sp. T92	10.40	
G1^*^	*Acinetobacter junii*	*Acinetobacter* sp.	24.30	*Acinetobacter* sp.	24.49	3.90
	*Acinetobacter johnsonii*					
	*Lactobacillus salivarius*	*Lactobacillus salivarius*	1.30	Family *Beijerinckiaceae*	26.40	0.001
	*Klebsiella pneumoniae*	*Klebsiella* sp. Z1	<0.01			
G2^*^	*Escherichia coli*	*Esherichia-Shigella*	2.80	*Bacteroides* sp.	17.23	10.96
				*Pseudomonas putida*	13.7	0.03
G3^*^	*Streptococcus canis*	*Streptococcus canis*	14.30	*Prevotella* sp. (COT 298)	31.10	15.21
				*Streptococcus canis*	14.30	6.68
	*Escherichia coli*	*Escherichia-Shigella*	10.80	*Escherichia-Shigella*	10.80	15.33
H	*Escherichia coli*	*Escherichia-Shigella*	1.70	*Acinetobacter* sp.	25.90	
				*Agrobacterium* sp. Emb7	13.80	
I1^*^	*Staphylococcus pseudintermedius*	*Staphylococcus pseudintermedius* E140	74.50	*Staphylococcus pseudintermedius* E140	74.50	1.52
	*Pseudomonas putida*	*Pseudomonas* sp.	1.50			
I2 (L)	*Achromobacter xylosoxidans*	*Achromobacter xylosoxidans*	6.20	*Acinetobacter* sp.	31.30	
				*Rhizobium* sp.	18.60	
I3 (R)	*Pseudomonas aeruginosa*	*Pseudomonas aeruginosa*	92.00	*Pseudomonas aeruginosa*	92.00	
	*Achromobacter xylosoxidans*	*Achromobacter xylosoxidans*	5.30			
J	*Bacteroides fragilis* (anaer.)	*Bacteroides* sp.	8.40	*Pseudomonas* sp.	20.30	
				*Brevundimonas* sp.	12.70	
				*Bradyrhizobium* sp. T92	12.50	
				*Acinetobacter* sp.	10.30	
K	No growth			*Pseudomonas putida*	71.90	
L	*Pseudomonas alcaligenes*	*Pseudomonas* sp.	8.50	*Pseudomonas putida*	30.20	
				*Alloprevotella* sp.	11.90	
M1^*^	*Klebsiella pneumoniae*	*Klebsiella* sp. Z1	34.80	*Klebsiella* sp. Z1	34.80	51.89
				*Acinetobacter* sp.	13.10	0.32
M2^*^	*Haemophilus parainfluenza*	*Haemophilus* sp. (COT 326)	6.40	*Prevotella* sp. (COT 298)	25.88	32.13
				*Bacteroides* sp.	15.77	12.48
N^*^	*Achromobacter* sp.	*Achromobacter xylosoxidans*	3.80	*Bradyrhizobium* sp. T92	57.10	1.08
	*Ochrobactrum anthropi*	Not detected				
	*Chryseobacterium* sp.	*Chryseobacterium* sp.	2.30			
O^*^	No growth			*Pasteurella* sp.	50.20	0.84
				*Pantoea* sp.	17.00	0.02

*Asterisks indicate dogs providing paired BALF samples and OP swabs*.

### DNA Extraction

To maximize yields, DNA was first extracted using a manual nucleic acid precipitation, followed by resuspension of DNA in buffer and purification using DNeasy kits (Qiagen) according to manufacturer's instructions with minor modifications. Briefly, BALF was first centrifuged at 5,000 × g for 10 min at room temperature, followed by removal of the supernatant and resuspension in 800 μL lysis buffer as adapted from Yu and Morrison (13). Samples were then incubated at 70°C for 20 min with periodic mixing and centrifuged as before. Next, 10 mM ammonium acetate (200 μL) was added to the supernatant and samples were incubated on ice for 5 min, before centrifugation at 5,000 × g for 10 min at room temperature. Up to 750 μL of the supernatant was then mixed with an equal volume of chilled isopropanol and incubated on ice for 30 min. Samples were then centrifuged at 16,000 × g for 15 min at 4°C. Precipitated nucleic acids were then washed with 70% ethanol, resuspended in 150 μL Tris-EDTA (10 mM Tris and 1 mM EDTA), and processed according to the DNeasy kit's manufacturer's instructions, with the following modification. Instead of eluting in the AE buffer provided with the kits, DNA was eluted in the comparable, but EDTA-free, EB buffer (Qiagen). Yields were determined via fluorometry (Qubit 2.0) using Qubit dsDNA BR assays (Life Technologies, Carlsbad, CA). Samples were stored at −20°C until library preparation was performed.

### 16S rRNA Library Preparation and Sequencing

Library construction and sequencing were performed at the University of Missouri DNA Core facility, as previously described (6). Briefly, 16S rRNA amplicons were generated via amplification of the V4 region of the 16S rRNA gene using single-indexed universal primers (U515F/806R) (15, 16) flanked by Illumina standard adapter sequences. Following amplification, products were pooled for sequencing using the Illumina MiSeq platform and V2 chemistry with 2 × 250 bp paired-end reads.

### Informatics

Assembly, annotation, and binning of DNA sequences were performed at the University of Missouri Informatics Research Core facility. Contiguous DNA sequences were assembled using FLASH software (17) and removed if found to be short after trimming for a base quality <31. Qiime v1.9.1 software (18) was used to perform *de novo* and reference-based chimera detection and removal, and remaining contiguous sequences were assigned to operational taxonomic units (OTUs) via *de novo* OTU clustering and a criterion of 97% nucleotide identity. Taxonomy was determined for selected OTUs using BLAST against the SILVA database (19, 20). Principal coordinate analyses (PCoA) were performed using ¼ root-transformed OTU relative abundance data in PAST 3.17 (21). Metrics of richness and α-diversity were determined based on a rarefied dataset subsampled to a uniform read count of 2,289 reads per sample using beta_diversity_through_plots.py, available at http://qiime.org/scripts/beta_diversity_through_plots.html.

### Statistical Analysis

Distribution of read counts in experimental and control samples was first tested for normality using the Shapiro-Wilk method, and differences in read count were then determined using a Mann-Whitney rank sum test due to non-normality, implemented in SigmaPlot 13.0. Differences in β-diversity between BALF and OP swab communities were determined using one-way permutational multivariate analysis of variance (PERMANOVA), implemented in Past 3.18 (21). To evaluate the similarity between OP and BALF bacterial communities in the context of SBP vs. CAP, time-matched OP and BALF samples were collected from a subset of patients (designated with a ^*^ in Table 2) and the intra-subject similarities between the OP and BALF communities in patients with SBP and CAP were visualized and tested for significance (between disease type) via principal coordinate analysis (PCoA) and one-way PERMANOVA, respectively. In both analyses (i.e., PCoA and PERMANOVA), comparisons were performed using both unweighted (i.e., Jaccard) and weighted (i.e., Bray-Curtis) metrics. Briefly, the Jaccard similarity is based on the agreement between two samples with regard to the proportion of shared taxa while the Bray-Curtis similarity also accounts for agreement between two samples with regard to the relative abundance of shared taxa. In all cases, significance was established as *p* < 0.05.

**Table 2:** Patient demographics related to samples included in the current analysis.

**Dog**	**Breed**	**Sex**	**Age**	**Wt. (kg)**	**Type of pneumonia**
A^*^	Siberian husky	MC	8 years	24.5	CAP
B^*^	Great Dane	F	6 months	25	CAP
C^*^	Great Dane	F	6 months	32.4	CAP
D	Giant Schnauzer	FS	1 year	25	CAP
E	Bulldog	MC	4 months	8	CAP
F	Great Dane	MC	5 years	71	CAP
G^*^	Mixed	MC	1 year	13.5	SBP; megaesophagus; AP
H^*^	Mastiff	FS	10 months	30	SBP; upper airway obstruction; AP
I^*^	Mixed	MC	8 years	8.8	SBP; chronic lower airway disease
J	Chesapeake Bay retriever	FS	4 years	31.7	SBP; chronic lower airway disease
K	Mixed	FS	1 year	10.7	SBP; pyothorax
L	Maltese	MC	6 years	9.8	SBP: tracheal FB; AP
M^*^	Welsh corgi	MC	2 years	8.6	SBP; tongue myopathy; AP
N^*^	Mixed	FS	10 years	13	SBP; UES achalasia; AP
O^*^	Border collie	MC	12 years	22	SBP; laryngeal paralysis; AP

MC, male castrated; FS, female spayed; CAP, community acquired pneumonia; SBP, secondary bacterial pneumonia; AP, aspiration pneumonia; FB, foreign body; UES, upper esophageal sphincter; Letters designated with

* *, paired OP and BALF samples*.

## Results

Twenty BALF samples were collected from 15 different dogs that met the enrollment criteria, i.e., a diagnosis of bacterial pneumonia based on clinical signs and associated with BALF septic suppurative inflammation or a positive culture result. Eighteen of those 20 samples had positive bacterial cultures, from which ten of 18 (56%) yielded one bacterial isolate, and eight of 18 (44%) yielded between two and five isolates.

Sequencing of 16S rRNA amplicon libraries generated from BALF and control fluid flushed through the bronchoscopes resulted in significantly different (*p* = 0.004) mean (± SEM) read counts of 31,524 (± 7,663) and 4,728 (±1,576) reads per sample, respectively. Additionally, seven BALF samples returning read counts within two standard deviations of the mean read counts generated from control samples showed generally good overall agreement with culture results indicating that the data were still meaningful. In contrast to the number of taxa identified based by culture, the DNA detected in patient BALF samples represented between 22 and 185 distinct operational taxonomic units (OTUs). Thus, while traditional culture methods provide evidence of live and cultivable bacteria in a sample and allow antimicrobial susceptibility testing, 16S rRNA sequencing provides a more comprehensive profile of taxa present in a sample, whether or not they are viable or cultivable. Additionally, mean ± SD numbers of unique OTUs in BALF from dogs with CAP were significantly lower than those with SBP (26 ± 16 and 82 ± 30 OTUs, respectively; *p* = 0.0002).

In many cases, there was remarkable agreement between the two methods, particularly in instances of a marked overgrowth of a dominant taxon. Specifically, samples from dogs A and D were both culture positive for *Streptococcus canis* alone, and 16S rRNA sequencing detected *S. canis* or *Streptococcus* sp. at 99.3 and 96.4% relative abundance (RA), respectively (Table 1). Similarly, samples I1, I3, and M1 were culture positive for *Staphylococcus pseudintermedius* and *Pseudomonas putida* (I1), *Pseudomonas aeruginosa* and *Achromobacter xylosoxidans* (I3), and *Klebsiella pneumoniae* (M1), with 16S sequencing detecting *S. pseudintermedius* (74.5% RA) and *P. putida* (1.5% RA), *P. aeruginosa* (92.0% RA) and *A. xylosoxidans* (5.3% RA), and *Klebsiella* sp. Z1 (34.8% RA), respectively in the same samples.

In the majority of the remaining samples, taxa detected via culture were also detected via sequencing but not as the dominant phylotype. In samples I2, M2, G1, G2, G3, H, J, L, F, and N, the dominant taxon determined by sequencing was not cultured despite many of the detected species being readily cultivable. In the aforementioned group of samples, cultured bacteria represented from <0.01 to 24.3% of the DNA detected via 16S rRNA sequencing (median 2.55% RA) or, in four of the eighteen culture-positive cases (22.2%), were not detected at all in the sequencing data (i.e., *Bordetella bronchiseptica* in samples F and H, *Enterococcus* spp. in sample G, and *Ochrobacterium anthropi* in sample N).

**Figure 1:**
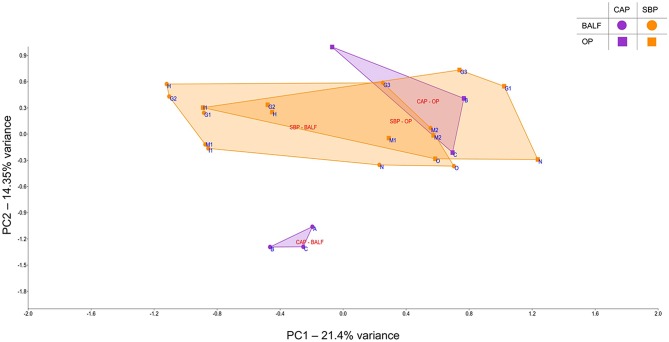
Principal component analysis of samples from bronchoalveolar lavage fluid (BALF) and oropharyngeal swabs (OP), for a select number of cases of community-acquired pneumonia (CAP) and secondary bacterial pneumonia (SBP); circles represent BALF, squares represent OP, SBP samples are in orange and CAP samples are in purple.

**Figure 2:**
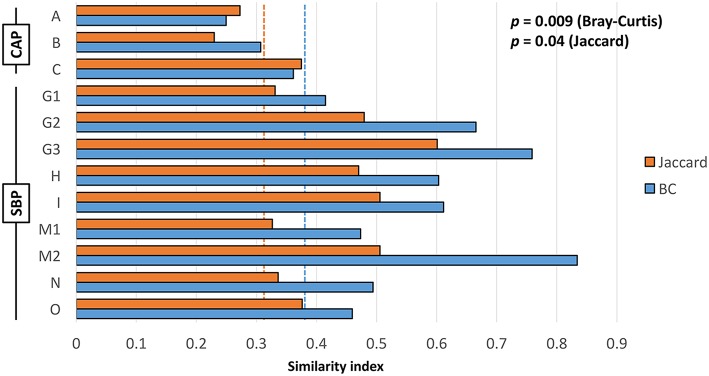
Intra-subject Jaccard (orange) and Bray-Curtis (blue) similarity between BALF and OP microbiota in cases of community-acquired pneumonia (CAP) and secondary bacterial pneumonia (SBP); dotted lines indicate mean inter-subject similarity between all samples. Results of Student's *t*-test shown on chart.

**Figure 3:**
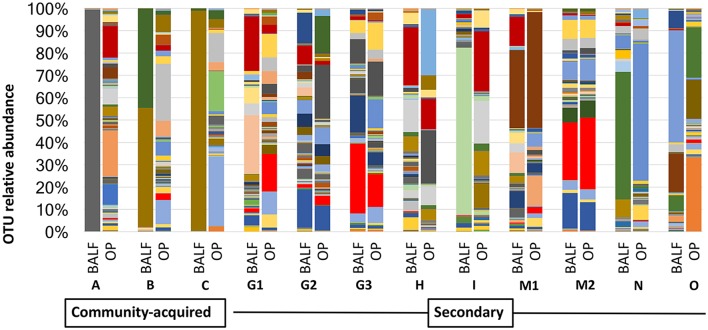
Comparison of relative abundance in BALF and OP between community acquired and secondary bacterial pneumonia.

In cases of SBP, we hypothesized that the same factors predisposing the dog to pneumonia would facilitate greater translocation of upper airway microbes to the lung, relative to what occurs in CAP. PCoA analysis revealed complete separation of BALF and OP samples in dogs with CAP, and substantial overlap between BALF and OP samples in dogs with SBP (Figure 1). Accordingly, PERMANOVA detected a significant difference between samples sites when based on the Jaccard similarity (*p* = 0.0003; *F* = 3.21), but not Bray-Curtis (*p* = 0.15; *F* = 1.32), indicating that the BALF and OP communities differ based on the presence or absence of certain taxa, but not with regard to the relative abundance of shared taxa. Factoring in individual variability, the intra-subject similarity between BALF and OP communities was low in the three cases of CAP from whom OP samples were collected (i.e., dogs A–C), regardless of the similarity index used (Figure 2). In contrast, samples from severely dysphagic dogs with confirmed aspiration pneumonia (e.g., dogs G and M) evinced a high degree of compositional similarity between BALF and OP microbiota (Figure 3). It is worth noting that, despite the apparent dissimilarity between BALF and OP swabs in samples from dogs diagnosed with CAP, the dominant taxa detected in the lower airways were consistently detected in the matched upper airway samples, albeit at a much lower relative abundance. Relative abundance (RA) of the dominant taxon is another metric used to describe resident bacterial communities in CAP and SBP. In 4/6 dogs with CAP, and in 1/9 dogs with SBP, there was near eradication of the microbial diversity in the lower airways, with predominant OTUs found in RAs between 92.4 and 99.9%.

## Discussion

Methods complementary to traditional laboratory-based culture of BALF in dogs with bacterial pneumonia, such as 16S rRNA sequencing, are useful to understand the complex relationship between pathogen and resident microbes. In this study, cultures of BALF, used by clinicians to provide insight into underlying pathogens, identified between zero and five bacterial species in dogs with bacterial pneumonia. This was in stark contrast to DNA sequencing that revealed rich microbial communities ranging from 22 to 185 distinct OTUs. Dysbiosis was appreciated by increases in the relative abundance of specific taxa, and interlinked with this change, a decrease in the predominating taxa found in healthy dog lungs (6). In the majority of BALF samples, the cultured bacteria were present in the sequencing data, despite variation between dogs as to whether the dominant taxa determined by culture was identical to the dominant taxa based on sequencing. This phenomenon has also been described in mechanically ventilated patients in which 75% of organisms identified via culture were the most abundant organisms identified via sequencing (22). An advantage of molecular techniques is their enhanced ability to provide insight as to how microbial communities change in disease and allow comparisons in regional differences in populations (i.e., upper vs. lower airways). Bacterial populations present in the lungs during pneumonia depend on the clinical scenario with differences between dogs with CAP and SBP. In the former, there was generally a stark loss of microbial diversity and replacement with a predominant taxon. In the latter, with secondary risk factors for pneumonia, such as laryngeal or esophageal dysfunction, lower airway communities are likely heavily derived from those present in the upper airways.

In a previous analysis of the lung microbiota of 16 healthy dogs at the same institution (6), the most abundant taxa were *Acinetobacter johnsonii, Brevundimonas diminuta* and members for the family *Pseudomonadaceae* [mean (range) % RA 19.81 (13.89–26.72), 22.48 (17.36–26.67), and 29.6 (18.57–34.94), respectively]. The remainder of the taxa identified in healthy dogs were present at a RA of 2.73% or less. This is in stark contrast to the findings in this study, where *Acinetobacter, Brevundimonas*, and members of the family *Pseudomonadaceae* were present between 10 and 50% RA in 9/20 samples, suggesting respiratory dysbiosis is a common component of canine bacterial pneumonia.

In dogs with bacterial pneumonia, cultures of BALF would seemingly support a single dominant or very small number (<5) of lung pathogens; however, results of DNA sequencing underscore the complexity of microbial communities in the lung, even in the presence of infection. Mean numbers of distinct OTUs identified in BALF of dogs with CAP (23) and SBP (82) were similar to a prior study of the healthy canine lower respiratory tract microbiome (6). Thus, richness (overall numbers of distinct OTUs), while variable in pneumonia, was discordant with the paucity of cultivable bacteria. Similarly, in a study evaluating respiratory microbial communities of mechanically ventilated patients with pneumonia, 12 out of 56 patients (21%) had positive cultures. These patients had a significant decrease in microbial richness compared to culture negative subjects there was a wide range in terms of richness (22). In patients that developed pneumonia after receiving lung transplantation also demonstrated a decrease in microbial richness (24). Most of the microbes found via sequencing are not cultivable using standard techniques, making it challenging to discern the larger picture of microbial interactions within the lung. It is unclear how many uncultured or as yet unidentified lung pathogens are responsible for bacterial pneumonia, but study of the respiratory microbiome will be critical moving forward.

The fact that, in 4 of 6 dogs with CAP, the predominant OTUs represented between 92.4 and 99.9% of recovered DNA suggests overgrowth of these organisms and reflect dramatic changes from that reported in healthy dogs (6). It has been proposed that disease is associated not just with pathogens gained, but with resident species that are lost and that a greater risk of repopulation with virulent organisms may be more likely with disappearance of dominant species associated with health (25). In dogs diagnosed with CAP, microbial diversity was abolished relative to dogs with SBP.

With regard to those bacteria identified via standard culture but not found in the sequencing data, *Bordetella bronchiseptica* is a common and important canine respiratory pathogen causing CAP that is also as a commensal organism in healthy, asymptomatic dogs (23, 26). It is unclear as to why *B. bronchiseptica* or closely related taxa were not detected in the 16S rRNA dataset. While the SILVA database used to annotate the current data does contain multiple sequences specific to other *Bordetella* spp., it is worth noting that the 16S rRNA sequence of *Achromobacter* and *Bordetella* spp. share a high degree of homology, and *A. xylosoxidans* was sequenced in both samples. It is also possible that the *Enterococcus* and *Bacillus* species identified via culture were a result of contamination, as these organisms are considered to be ubiquitous. There are clinical implications to identification of organisms on culture that are not present in the sample according to targeted sequencing. In large part, results of standard culture techniques are relied upon for information to guide treatment. This could potentially lead to incorrect antibiotic selection.

Conversely, there are also limitations to the utility of molecular approaches, the most apparent of which is the inability to distinguish viable and dead bacteria using standard 16S rRNA sequencing methods. The fact that many of the bacterial species found in the sequencing data were also cultured indicates that at least some of the bacteria present in the lungs are viable, and even if DNA detected in BALF represents bacteria that have been killed by the immune defenses of the lung (e.g., pulmonary alveolar macrophages), it does not obviate an influence of those bacteria on airway health. Without a viable isolate, it is also not possible to perform antimicrobial susceptibility testing on taxa identified via sequencing. Moreover, whether applying traditional culture-based or culture-independent molecular techniques, there is an inherent risk of contamination and identification of false positives. However, while a single CFU can theoretically be detected on culture, the competitive nature of 16S rRNA sequencing (i.e., an overabundance of DNA amidst a limited number of binding substrates on the sequencer flow cell) renders single CFUs as background noise and inadvertent contaminants will not be detected as dominant taxa in the data. Lastly, clinicians are limited by our still nascent appreciation of these microbial communities. Until there is a better understanding of the lung microbiota in the context of both health and disease, it will be challenging to incorporate sequencing data into clinical decisions.

The development of targeted sequencing techniques highlights limitations associated with standard culture. The belief that the lower airways are sterile is still perpetuated in textbooks; however, the notion of lung sterility has been refuted by a number of studies following the first culture-independent report of the healthy human lung microbiome (27). Healthy dogs and other species have diverse and dynamically changing microbial communities inhabiting specific ecologic niches in the lower airways in the absence of clinical evidence of infection (6, 28–31). The composition of the lung microbiome depends on microbial immigration into the airways, microbial elimination and the relative reproduction rates of host microbial communities determined by regional growth conditions (32). Immigration is influenced by local mucosal extension from the upper airways that lack a physical barrier separating them from the lower airways, microaspiration, and inhalation of bacteria from ambient air (8, 33). Protective reflexes, such as cough, mucociliary function, and innate and adaptive mucosal immune responses affect elimination (8). Although bacterial pneumonia has been regarded as resulting from invasion and growth of a pathogen in the lungs, recent work suggests a primary driver of disease is disruption of homeostasis of the complex microbial ecosystem (34). The upper respiratory tract has been called the gatekeeper to respiratory health, wherein “colonization resistance” is provided by local bacterial communities preventing establishment of mucosal infections capable of spreading to the lower respiratory tract. Thus, while the airway microbiome has the capacity to blunt growth of pathogenic species during states of equilibrium, dysbiosis of upper airways in humans has been linked to CAP (35), SBP (36), and ventilator associated pneumonia (22). In the subset of dogs with paired OP and BALF samples, 3 dogs had CAP, and 8 had SBP. The dogs with CAP were more likely to have a single OTU predominate. In contrast, dogs with SBP were more likely to have ≥2 OTUs. It is interesting to note that in several cases (G3, M1, M2), one of the OTUs found in BALF at a relative abundance of >10% was also present in the OP at an even greater relative abundance. Taking into consideration an underlying process disrupting homeostasis in SBP, it was not surprising that dogs with SBP had more similar microbial compositions of the upper and lower airways (i.e., more evident regional continuity) and greater diversity than dogs with CAP.

While traditional culture methods provide evidence of live and cultivable bacteria in a sample and allow antimicrobial susceptibility testing, 16S rRNA sequencing provides a more comprehensive profile of taxa present in a sample, whether or not they are viable or cultivable. Additionally, these data highlight that just as lack of growth does not imply a sterile environment, identification of a particular organism with either approach does not imply that this organism is the causative agent of disease (37). Lack of growth could also be related to the presence of fastidious organisms, such as *Mycobacterium* that require specific media or growth conditions using standard methods as it has been documented in people with pneumonia (38) Bacterial culture with sensitivity testing guides treatment of clinical infections and thus will still play a key role in therapy of bacterial pneumonia. However, culture-independent techniques may provide greater depth of understanding of the changes in microbial composition that occur in bacterial pneumonia. These methods could allow for identification of pathogens that may not be readily cultivable, help discriminate true pathogens from colonizing bacteria (37) and provide insight into potential treatment strategies that restore balance toward a microbial population associated with health.

## Conclusions

In comparing standard culture and targeted sequencing techniques to identify organisms found in BALF of dogs with bacterial pneumonia, we demonstrated discrepancies between these techniques in terms of presence or absence of predominating taxa and numbers of unique bacteria. Dysbiosis of the respiratory microbiome is a key feature of canine pneumonia, with decreased relative abundance of bacterial community members found in health. Additionally, there appears to be greater regional continuity between the upper and lower airways in dogs with SBP. While much more commonly observed in dogs with CAP than SBP, obliteration of microbial diversity with evidence of overgrowth of one organism was noted in one-third of the dogs in this study. This may suggest that loss of dominant species associated with health could underlie disease pathology. Clinical application of DNA sequencing may be employed if culture is negative in dogs with compatible clinical signs and septic suppurative BALF cytology, or if targeted antimicrobial therapy against the cultivable bacteria fails to produce disease resolution. Future studies aimed at restoring a dysbiotic airway microbiome in canine bacterial pneumonia are warranted.

## Data Availability Statement

All sequence data have been deposited in the NCBIA Sequence Read Archive (SRA) under the BioProject accession number: PRJNA510415.

## Ethics Statement

All studies were performed in accordance with the Guide for the Use and Care of Laboratory Animals, and were approved by the University of Missouri Institutional Animal Care and Use Committee (MU IACUC protocol # 8240).

## Author Contributions

AV-P participated in the conception and design of the study, sample collection, DNA extraction, data interpretation, and co-authored the manuscript. AE participated in the conception and design of the study, contributed resources, interpreted sequence data, and co-authored the manuscript. CR participated in conception and design of the study, sample collection, data interpretation, and co-authored the manuscript. HR assisted with DNA extraction, sample collection, and study coordination. All authors read and approved the final manuscript.

### Conflict of Interest

The authors declare that the research was conducted in the absence of any commercial or financial relationships that could be construed as a potential conflict of interest.

## Abbreviations

BALF: bronchoalveolar lavage fluid
CAP: community-acquired pneumonia
OUT: operational taxonomic unit
PCoA: principal coordinate analysis
RA: relative abundance
SBP: secondary bacterial pneumonia.
